# Immunoinformatic design of a multivalent vaccine against *Brucella abortus* and its evaluation in a murine model using a DNA prime-protein boost strategy

**DOI:** 10.3389/fimmu.2024.1456078

**Published:** 2024-11-21

**Authors:** Raúl E. Molina, Alberto Osorio, Manuel Flores-Concha, Leonardo A. Gómez, Ilse Alvarado, Italo Ferrari, Angel Oñate

**Affiliations:** ^1^ Laboratory of Molecular Immunology, Department of Microbiology, Faculty of Biological Sciences, University of Concepción, Concepción, Chile; ^2^ Simes Educational Center, Santiago, Chile

**Keywords:** prime-boost strategy, multivalent vaccines, immunoinformatic, molecular dynamics, immunogenicity, protection, interferon gamma

## Abstract

**Introduction:**

The development of effective vaccines against *Brucella abortus* is critical due to its significant impact on human and animal health. The objective of this study was to design and evaluate *in silico* and *in vivo* a multivalent vaccine based on the immunogenic potential of three selected open reading frames (ORFs) of *Brucella*.

**Methods:**

The designed construct, named S22, was analyzed *in silico* to evaluate its physicochemical properties, antigenicity, allergenicity and toxicity. This construct was modeled and subjected to molecular dynamics analysis. Additionally, the antigenicity and protection induced by this construct was evaluated through *In vivo* assays immunizing BALB/c mice with protein (S22), DNA (pVS22) and combining both vaccine formats using a prime boost immunization strategy.

**Results:**

All bioinformatics analyses showed safe and high quality structural features, revealing favorable interactions between S22 and the TLR4/MD2 complex. Moreover, results from *in vivo* assays indicated that the S22 protein induced robust levels of IgG1 and IgG2a, suggesting a balanced Th1 and Th2 immune response. The DNA construct (pVS22) elicited primarily a Th1 response, whereas the use of a prime boost strategy, which combines both formats resulted in a balanced immune response with significant induction of lymphoproliferation and elevated.

**Discussion:**

Although our assays did not demonstrate the induction of a substantial protective response against *B. abortus*, this construct was capable of inducing immunogenicity. This study highlights the utility of *in silico* design for predicting and optimizing candidate vaccines and underscores the potential of using strategies such as prime boost, which incorporate antigens of different biological nature to modulate the immune response, while balancing parameters such as stability of the antigens and the cost of production.

## 1 Introduction

The genus *Brucella* comprises small, non-motile, and non-sporulating Gram-negative coccobacilli. These facultative intracellular pathogens lack classical virulence determinants such as capsules and plasmids (1). *Brucella* spp are known for their ability to infect mammalian cells and establish chronic infections in both wildlife and domestic animals, as well as in humans, leading to brucellosis. In cattle, *Brucella abortus* causes sterility in males and abortion in pregnant females. In humans, this bacterium is responsible for a zoonotic disease primarily acquired through contact with infected animals or consuming contaminated dairy products. Human brucellosis can progress to a chronic condition with symptoms like intermittent fever, myalgia, and headache, potentially escalating to hepatitis, osteoarthritis, endocarditis, and neurobrucellosis (2). This chronic condition is attributed to the tropism of *B. abortus* for the lymphoreticular and reproductive systems, facilitating its survival within phagocytic cells and evasion of the host’s innate and adaptive immune responses (3).

Upon infection, *B. abortus* evades the microbiocidal action of the complement system and the recognition by the pathogen-associated pattern recognition receptors (PPRs), thereby limiting Toll-like receptor (TLRs) signaling pathways and innate functions of several leukocytes (4). The stealthy nature of *B. abortus* has been largely attributed to the smooth structure of its lipopolysaccharide (Br-LPS). The Br-LPS, with an elongated fatty acid in the lipid A component, exhibits low toxicity and a reduced capacity to activate the immune response by evading TLR4 recognition (5). *B. abortus* also expresses immunoregulatory elements such as BtpA and BtpB proteins, which translocate through the type IV secretion system (T4SS), inhibiting TLR signaling in innate cells and dampening the adaptive immune responses. BtpA specifically inhibits TLR2 and TLR4, while BtpB is a potent inhibitor of TLR2, TLR4, and TLR9 signaling (6, 7). This stealth strategy challenges the induction of protective immunity, which is dependent on cellular immunity mediated by CD4^+^ T helper type 1 and CD8^+^ T cells (8).

Vaccination remains a principal method for preventing, controlling, and eradicating brucellosis. Currently, two live attenuated vaccines based on *B. abortus* strain 19 (S19) and *B. abortus* strain RB51 are widely used in preventing bovine brucellosis (9, 10). Despite their effectiveness, these vaccines can induce adverse effects in cattle, such as abortion, and pose a risk to humans, with the RB51 strain showing resistance to rifampicin, a primary antibiotic used to treat human brucellosis (11, 12). Furthermore, the administration of the *B. abortus* S19 strain, due to its LPS, generates diagnostic problems, since it does not allow differentiating between immunized animals and those that are naturally infected (13). These safety concerns underscore the urgent need for safer vaccine alternatives.

Recent vaccine candidates against brucellosis have shown promising results, including DNA vaccines encoding genes for lumazine synthase (BLS) (14), Cu/Zn superoxide dismutase (SOD) protein (15), and various open reading frames (ORFs) such as BAB1_0267, BAB1_0270, BAB1_0278, BAB1_0278a, as well as a multiepitope DNA vaccine designed using immunoinformatic approaches (16). Despite their demonstrated immunogenicity and significant protection levels in mice infected with *B. abortus* 2308, these vaccine candidates exhibit considerable variability in protection levels. This observation was described by the BAB1_0267 and BAB1_0270 ORFs coding for a ZnMP and a SH3-like domain-containing protein, respectively, which are DNA vaccines that induced significant levels of IgG antibodies, cytokines (IFN-gamma) and lymphoproliferative responses; however, they conferred low levels of protection (17). Therefore, the prime-boost immunization strategy, either with the same (homologous prime-boost) or a different formulation (heterologous prime-boost), has emerged as an effective approach to enhance the immunogenicity of DNA vaccines (18). Notably, the DNA prime-protein boost approach has successfully induced both humoral and cellular immune responses against several pathogens (19), suggesting its potential applicability in enhancing the immunogenicity of DNA vaccine candidates against *B. abortus*. In this study, we aim to contribute to developing an effective and safer vaccine against *B. abortus.* Utilizing bioinformatics tools, we designed a multivalent DNA vaccine and a homologous recombinant protein vaccine based on Cu/Zn SOD, BAB1_0270 ORF (ZnMP), and BAB1_0267 ORF (SH3-like domain) proteins from *B. abortus* 2308. We evaluated the immunogenicity and protective response conferred by these vaccines in BALB/c mice, both administered alone and through a DNA prime-protein boost strategy.

## 2 Materials and methods

### 2.1 An *in silico* design of the multivalent protein and its physicochemical and immunological parameters was used

The multivalent protein, designated S22, was designed using amino acid sequences from *B. abortus* strain 2308, which is available in the NCBI GenBank database. The S22 protein incorporates sequences of the ZnMP protein (Accession number: CAJ10226), SH3-like domain protein (Accession number: CAJ10223), and the SOD protein (Accession number: CAJ12701), linked by a (GGGGS)^4^ peptide. The physicochemical properties of the S22 protein were analyzed using the Expasy ProtParam tool (20). Its solubility was evaluated using the SOLpro server (Magnan et al., 2009). Immunological parameters, such as antigenicity, were assessed using VaxiJen v2.0 and ANTIGENpro (21), while the allergenicity and toxicity were evaluated using AllerTOP v.2.0 and ToxinPred2 servers, respectively (22, 23).

### 2.2 Tertiary structure modeling and molecular docking of the multivalent protein

The tertiary structure of the S22 protein was predicted using the IntFold7 server (24) and refined with the GalaxyRefine web server (25). Model quality was evaluated using ProSA-web (26), and stereochemical quality was validated using the MolProbity server (27). Molecular docking was performed with ClusPro 2.0 (28), using the crystal structure of the TLR4/MD-2/lipid IVa complex from *Mus musculus* (PDB accession number: 3VQ1) (29). For adjuvating functions, one chain of Lymphocyte antigen 96 and one of the Toll-like receptor 4 were selected and docked against the S22 construct using ClusPro 2.0 server (30).

### 2.3 Molecular dynamics simulations

The stability of S22, both alone and in complex with TLR4, was confirmed through Molecular Dynamics Simulations (MDS). CHARMM-GUI server’s Solution Builder tool constructed the input for GROMACS, using the TIP3P water model for both MDS setups. Monte Carlo methods were used to add K^+^ and Cl^-^ ions to each system until neutralization (31). The Amber Force Field ff19sb was used for the system description (32). System potential energy minimization employed the steepest descent algorithm. The NVT and NPT ensembles were applied at 300K and 1 bar. The final MDS, with a 2 fs integration time step, was conducted for 45 ns. Energy recordings were made every 10 ps. The conformational stability of S22 and the S22-TLR4 complex was assessed by generating RMSD (root mean square deviation) profiles for backbone residues, RG (radius of gyration), and RMSF (root mean square fluctuation) from C-alpha atoms.

### 2.4 Production of multivalent recombinant protein

The S22 gene was synthesized by GenScript (Piscataway, NJ, USA) with a 6-his tag at the carboxy terminal. The recombinant S22 protein was expressed and purified in *E. coli* by Novoprotein Scientific Inc. (New Jersey, USA), whose description is that it would have ≥85% purity, with an endotoxin level of 1 EU (endotoxin units)/μg of protein, using for this detection a polymyxin B bead column (Polymyxin Affi-Prep; Bio-Rad Laboratories, Hercules, California).

### 2.5 Construction and purification of the multivalent DNA vaccine

The gene for the S22 expression in eukaryotic cells was synthesized and codon-optimized by GenScript (Piscataway, NJ, USA) with a Kozak sequence addition. It was then subcloned into the pVAX eukaryotic expression vector (Thermo Fisher Scientific Inc., USA), resulting in the construct pVS22, used as a DNA vaccine. Large-scale plasmid purification followed the method described by (33).

### 2.6 Evaluation of immune response

#### 2.6.1 Animals

Animal experiments received approval from the ethics, bioethics, and biosafety committee of the University of Concepción, certificate CEBB 1466-2023. Female BALB/c mice, aged 10 weeks, were obtained from the Institute of Public Health (ISP), Santiago, Chile. Mice were randomly distributed into groups of five and housed with food and water ad libitum at the Molecular Immunology Laboratory, Department of Microbiology, University of Concepción.

#### 2.6.2 Schedule of immunization

**Figure 1:**
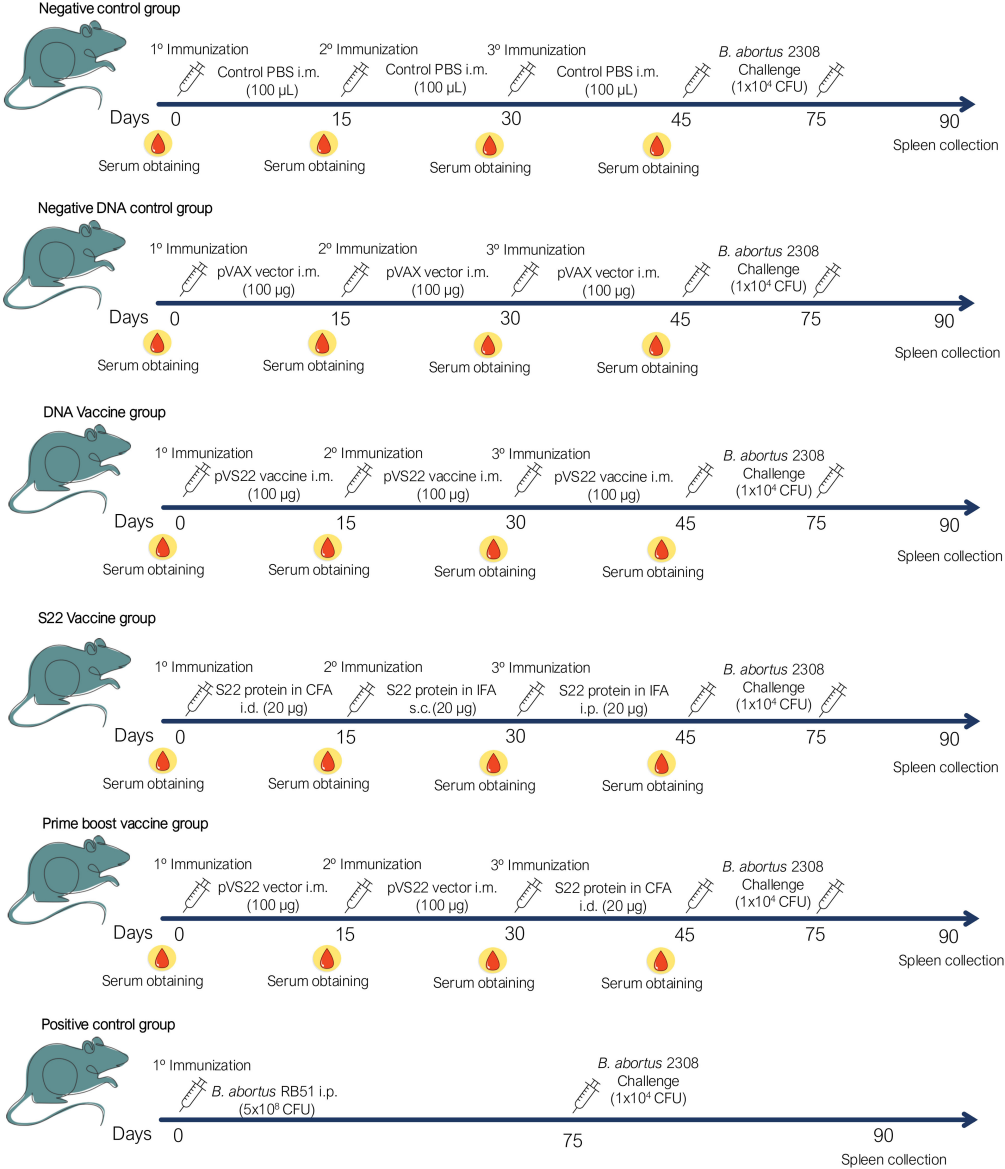
Schematic representation of the immunization and challenge protocol. Each group received three doses of their respective vaccines or PBS in a final volume of 100 µL. In addition, a positive control group was included, which was immunized once intraperitoneally (i.p.) with 5 x 10^8 CFU of *B. abortus* RB51. After 45 days from the last immunization, mice in each group were challenged with 1 x 10^4 CFU of *B. abortus* 2308. 15 days after challenge, the mice were euthanized and spleen were collected to evaluate the protection of each immunization strategy. Routes of administration: i.m. (intramuscular), i.d. (intradermal), s.c. (subcutaneous) and i.p. (intraperitoneal).

The BALB/c mice were immunized with either the recombinant multivalent protein S22, the plasmid pVS22 DNA vaccine, or both in a DNA prime-protein boost strategy, administered at 15-day intervals. One group received an initial intradermal immunization with 20 µg of S22 (50 µl) emulsified in 50 µl of Freund’s Complete Adjuvant (FCA) (Sigma), followed by subcutaneous and intraperitoneal administrations with 20 µg of S22 (50 µl) emulsified in 50 µl of Freund’s Incomplete Adjuvant (FIA) (Sigma). Another group received three intramuscular immunizations with 50 µg of the pVS22 DNA vaccine in 50 µl of PBS in each tibialis anterior muscle (100 µg of DNA/mouse). For the prime-boost strategy, the initial two immunizations were administered intramuscularly with 20 µg of pVS22 DNA vaccine, followed by an intradermal dose of 20 µg S22 in FCA. The control groups received either the pVAX empty vector (100 µg of DNA/mouse) or 100 µl of sterile PBS through intramuscular injections. A positive control group received a single intraperitoneal dose of 5 x 10^8 CFU of the *B. abortus* RB51 vaccine (**Figure 1**). Serum samples were obtained from blood drawn from the caudal tail vein two days before each immunization and were stored at -20°C until use (**Figure 1**).

#### 2.6.3 Antibody response

Antibody levels against S22 protein were measured in sera using an indirect ELISA. Briefly, 96-well plates were coated with 3 μg/ml of S22 protein diluted in carbonate-bicarbonate buffer (pH 9.4) and incubated overnight at 4°C. After the plate was washed three times (3X), serial dilutions of the serum, starting at 1:200, were added. Plates were incubated for three hours at room temperature, followed by three washes with PBS.Anti-S22 IgG1 and IgG2a were detected using horseradish peroxidase (HRP)-conjugated rat anti-mouse IgG1 (US Biological Life Sciences, Salem, MA, USA) and HRP-conjugated goat anti-mouse IgG2a (Jackson ImmunoResearch Inc., West Grove, PA, USA), respectively. Both secondary antibodies were diluted 1:3000 and incubated for 30 minutes at room temperature. Finally, the reaction was measured through the colorimetric response of o-phenylenediamine dihydrochloride (Sigma-Aldrich) with HRP, read at 450 nm using an Infiniti M Nano instrument (TECAN Group Ltd. Switzerland) (34).

#### 2.6.4 Lymphoproliferative response

Forty-five days post-immunization, mice were euthanized to harvest spleens. Spleens were disaggregated to prepare cellular suspensions, and red blood cells were lysed using AcK solution (Promega, USA). After washing the splenocytes thrice with PBS, they were adjusted to 4 x 10^6 cells/mL in RPMI-1640 supplemented with fetal bovine serum and antibiotic-antimycotic solution (Sigma). Each well of a 96-well cell culture plate received 100 µl of splenocytes. Later, the cells were stimulated by adding 100 µl of S22 or total proteins from *B. abortus* at concentrations of 0.5, 2.5, and 12.5 µg/ml. The cultures were incubated for 72 hours at 37°C in a 5% CO2 atmosphere. After this, the proliferation assay involved a pulse with 0.5 µCi/well of [Methyl-3H] thymidine for 8 hours, followed by harvesting and assessing the incorporated thymidine (c.p.m) using a scintillation counter. Concanavalin A (Merck) at a concentration of 10 µg/ml was used as a positive control, and a complete RPMI 1640 medium was used as a negative control. All assays were performed in triplicate.

#### 2.6.5 Production of cytokines

Levels of cytokines produced by T helper lymphocytes were determined in the supernatants of splenocyte cultures. Briefly, 500 µL of splenocytes (4 x 10^6 cells/ml) were stimulated with 500 µL of S22 at different concentrations. These cultures were incubated in 24-well plates for 72 hours at 37°C in a 5% CO2 atmosphere. After incubation, supernatants were collected for cytokine quantification using sandwich ELISA. The production of IFN-gamma, TNF-alpha, and IL-4 by splenocytes was measured using the Invitrogen ELISA kit (Thermo Fisher Scientific Inc., USA). Standard curves were established for cytokine quantification (34, 35). Finally, absorbance generated by HRP enzyme-binding secondary antibodies was read at 450 nm using the Infiniti M Nano (TECAN Group Ltd. Switzerland). All assays were performed in triplicate.

#### 2.6.6 Challenge assays

The protective efficacy of the vaccine formulations, including a positive control group immunized with 5x10^8 CFU of *B. abortus* RB51, was evaluated 45 days after the last immunization (**Figure 1**). For this, experimental and control mice were intraperitoneally challenged with 1 x 10^4 CFU of *B. abortus* 2308. Then, fifteen days post-challenge, mice were euthanized, and their spleens were aseptically removed and homogenized in 2 ml of sterile PBS. The homogenates were serially diluted and cultured through microdrops on Columbia agar plates supplemented with 5% sheep blood (bioMériex, France). After 72 h of incubation at 37°C, colonies were counted to determine each animal’s CFU count per spleen. The level of protection was quantified subtracting the log10 of CFU counts from spleens of unimmunized (negative control) and log10 of immunized mice (34). All assays were done in duplicate.

### 2.7 Statistical analysis

Statistical analyses were performed using the GraphPad Prism 9 software (GraphPad Software Inc., La Jolla, CA, USA). A two-way ANOVA evaluated data from antibody response, lymphoproliferative response, and cytokine production assays. *Post hoc* analyses identified specific group differences using Tukey’s multiple comparison test. A one-way ANOVA was employed for the protection assay data analysis, followed by Tukey’s test for multiple comparisons.

## 3 Results

### 3.1 Secondary structure, physicochemical, and immunological parameters of the vaccine to be developed

**Figure 2:**
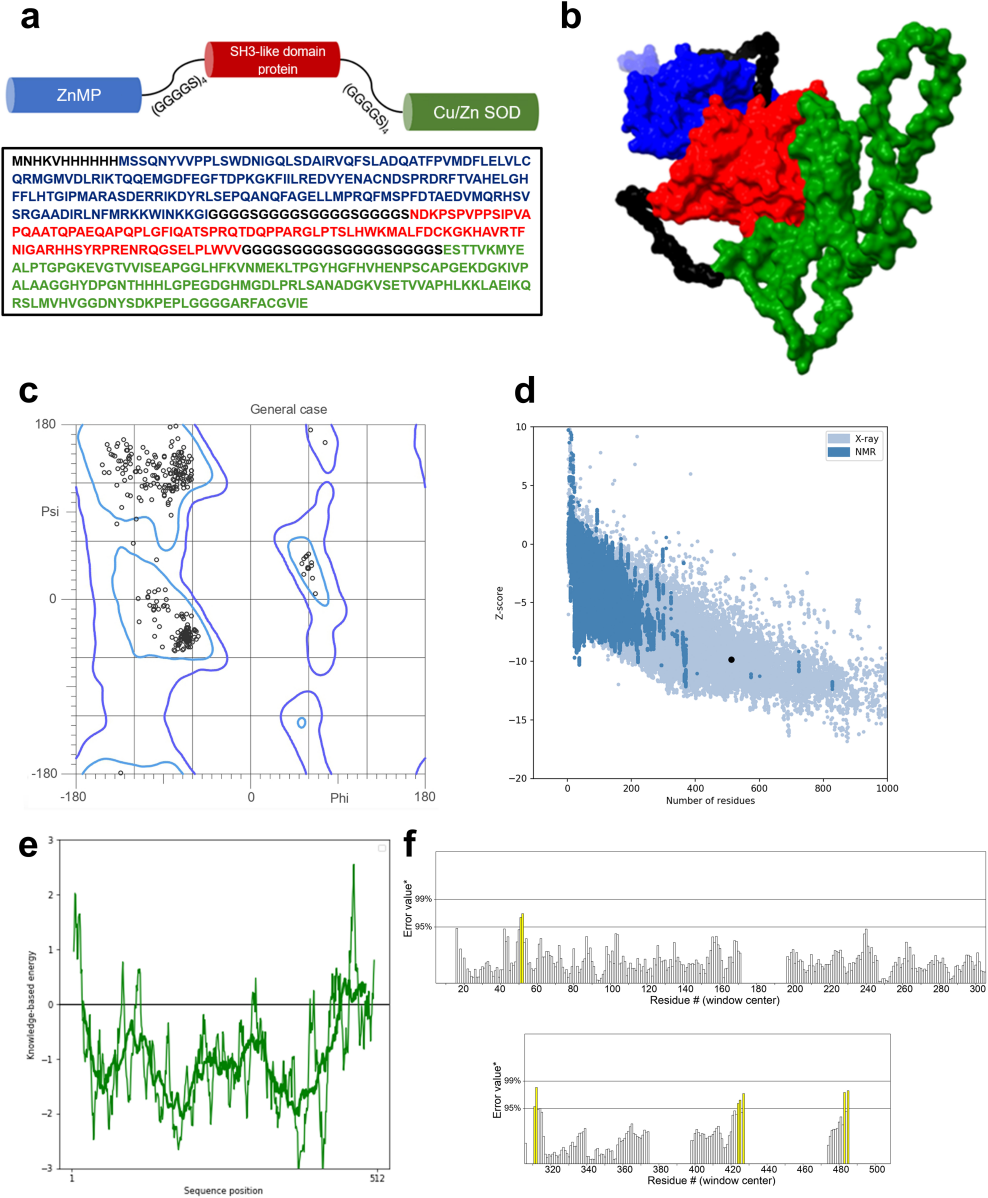
**(A)** Aminoacidic sequence of the designed protein and schematic representation of the proposed model. **(B)** Refined 3D model of the designed vaccine obtained from the IntFold7 server and refined by the GalaxyRefine server. Blue protein SOD, red protein ZnMP, and in green, the SH3-like domain protein. **(C)** Ramachandran plot of the refined model indicates that 98% of the residues are in favored regions and 99.8% of residues are in allowed regions. **(D)** ProSA-web overall model quality plot displaying the model Z-score of -9.85. **(E)** ProSA-web local quality model showing average energies calculations in 40 residues window. **(F)** ERRAT plot showing an overall quality factor of 97.554. Yellow bars represent regions with an error rate between 95% and 99%.

We designed a multivalent vaccine candidate, called S22, based on the amino acid sequences of three distinct proteins. The S22 structure encompasses the Cu-Zn SOD protein at the N-terminus, followed by the zinc-dependent metallopeptidase (BAB1_0270), and the SH3 domain-containing protein (BAB1_0267) at the C-terminus. The flexible linker (GGGGS)_4_ connected these proteins (**Figure 2A**). As a result, the S22 fusion protein consists of 512 amino acids with a molecular weight predicted of 54.47 kDa. Regarding its physical and chemical properties, it was determined that S22 has a theoretical isoelectric point of 8.18, an instability index of 46.72, an aliphatic index of 67.09, and a GRAVY (Grand Average of Hydropathy) value of -0.354, indicating its hydrophilic nature. Solubility predictions using the SOLpro server suggest that S22 is soluble, yielding a solubility probability score of 0.545. Regarding immunogenicity, the VaxiJen v2.0 server classified S22 as a probable antigen with a score of 0.967, above the threshold of 0.4 for bacterial proteins. ANTIGENpro predicted a 0.801 probability of antigenicity. Additionally, the AllerTOP v.2.0 server identified S22 as a likely non-allergen, and the ToxinPred2 server classified it as non-toxic with a Hybrid Score of -0.31 (**Table 1**).

**Table 1:** The physicochemical properties, immunological parameters and docking score of the designed vaccine construct.

Tool/Parameter	Value
Number of Amino Acids	512
Molecular weight (Daltons)	54kDa
GC-content	69.27%
Theoretical pI	8.18
^a^Instability index	46.72
Aliphatic index	67.09
Hydropathicity GRAVY	-0.354
^b^SoLpro	0.545
Antigenicity (VaxiJen 2.0 scores)	0.967
Antigenicity (ANTIGENpro scores)	0.801
Allergenicity	Non-Allergen
Toxicity (ToxinPred2 scores)	Non-Toxin - Hybrid Score of -0.31
Binding Energy	-16.3 ΔG(kcal/mol)

a The instability index provides an estimate of the stability of a protein in a test tube. The instability index values for proteins range from 18.43 to 45.31.

b Scaled solubility value (0–1). A values greater than 0.45 predicts that the protein is soluble.

### 3.2 Tertiary structure prediction and validation

The tertiary structure of S22 was modeled using the IntFold7 server. The selected model, chosen for its high confidence and global model quality score of 0.52, is depicted in **Figure 2B**. Further refinement with GalaxyRefine resulted in a model validated by a MolProbity score of 1.23. Ramachandran analysis revealed that 98% of amino acid residues were in favored regions and 99.8% in allowed regions (**Figure 2C**). ProSA-web assessment yielded an estimated Z-score of -9.85, which is within the range of scores found for native proteins of similar size (**Figure 2D**). Local quality estimation indicated predominantly average energy calculations below zero in 40-residue windows (**Figure 2E**). Lastly, the ERRAT analysis, assessing overall quality, showed a high score of 97.5 (**Figure 2F**).

### 3.3 Docking and molecular dynamics analysis

**Figure 3:**
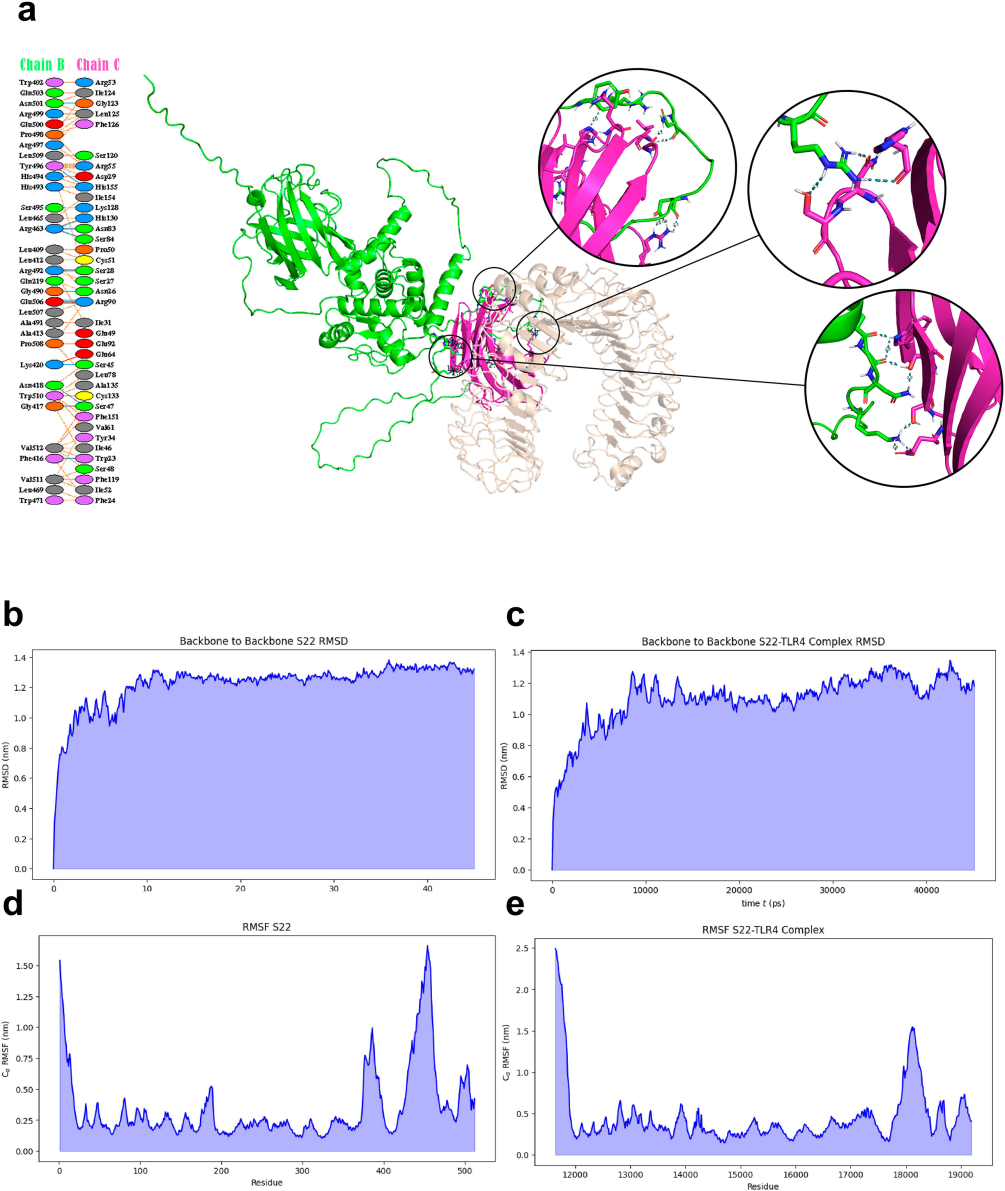
**(A)** Molecular docking complex representation of S22 protein vaccine and TLR4 complex. Interacting residues between the S22 protein and TLR4/lymphocyte antigen 96 complex are highlighted. **(B–E)** MD simulation trajectory-based graphs for analysis of structural stability. Graphs generated by GROMACS at different stages of MD simulations of the designed construct.

The interaction between the S22 protein and TLR4 was evaluated using docking and molecular dynamic analysis. The PDBsum server revealed interactions of the S22 with LY96, including 14 hydrogen bonds and 2 salt bridges between S22 and LY96, chains B and C, respectively (**Figure 3A**). The PRODIGY server calculated a binding energy value of -16.3 ΔG (kcal/mol), indicating a strong interaction. Molecular dynamics simulation revealed a stabilization of the S22 at 15 ns in both free form and when complexed with TLR4, although the latter conformation showed less consistency (**Figures 3B, C**). RMSF analysis indicated no unusual fluctuation in S22. The free form exhibited higher fluctuations at the (GGGGS)_4_ linkers and the SH3 domain-like protein (BAB1_0267) (**Figure 3D**), while the S22-TLR4 complex showed reduced fluctuations, especially in the linker’s regions (**Figure 3E**).

### 3.4 Analysis of the recombinant protein S22 and the plasmids pVS22 used for immunization

**Figure 4:**
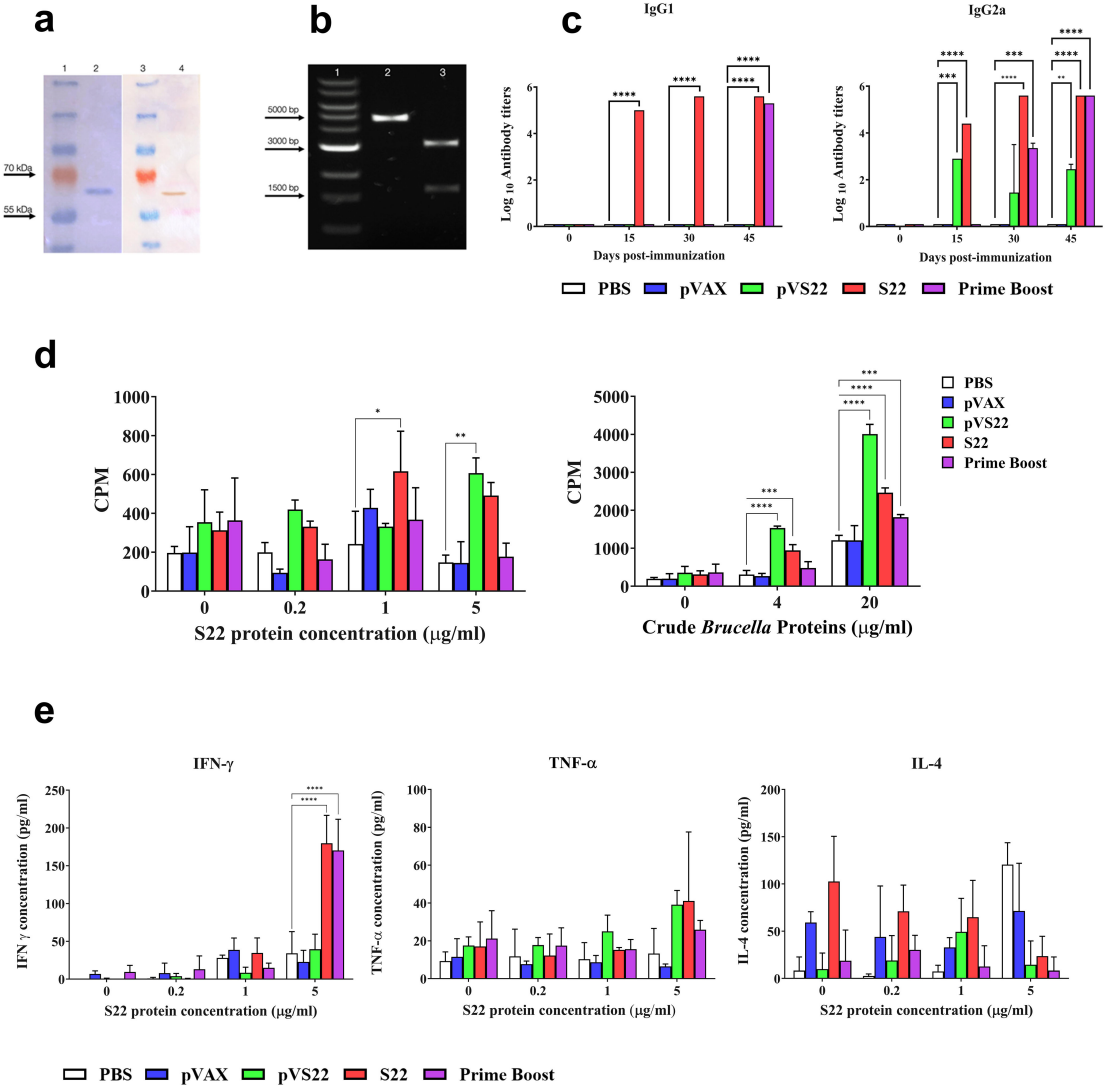
**(A)** SDS PAGE and Western blot analysis of the purified protein. Lane 1: Protein molecular weight marker. Lane 2: S22 protein SDS PAGE electrophoresis band. Lane 3: Protein molecular weight marker. Lane 4: Immunodetection of 6xHis Tag of S22 protein by Western blot. **(B)** Agarose gel electrophoresis of the pVS22 plasmid. Lane 1: DNA molecular weight marker. Lane 2: Linearized pVS22 plasmid. Lane 3: Products of the *PstI* and *BamHI* restriction enzymes double digestion of pVS22 plasmid. **(C)** Serum IgG1 and IgG2a antibody titers measured by ELISA at days 0, 15, 30, and 45 post-immunizations. Results are expressed as mean ± SD of log_10_ of the last reciprocal serum dilution value above the cut-off. **(D)** Lymphoproliferative response of splenocytes after *in vitro* stimulation with 0, 0.2, 1, or 5 μg/ml of recombinant S22 protein, and 0, 4, or 20 μg /ml of *B. abortus* 2308 total proteins (crude *Brucella* proteins). Results are shown as mean ± SD of ^3^H-thymidine incorporation from cells (CPM, counts per minute). **(E)** Cytokine levels of IFN-γ, IL-4, and TNF-α quantified by sandwich ELISA from the supernatant of *in vitro* stimulated splenocytes with 0, 0.2, 1, or 5 μg /ml of recombinant S22 protein. Results are expressed as mean ± SD of cytokine concentration. * P < 0.05; ** P < 0.01; *** P < 0.001 and **** P < 0.0001.

The recombinant multivalent protein S22, supplied by Novoprotein Inc., was analyzed for purity using SDS-PAGE electrophoresis. The results showed a single band of approximately 65 kDa, corresponding to the recombinant protein’s molecular weight plus the histidine tail (**Figure 4A**, lane 2). A Western blot analysis using antibodies against the 6xHistag confirmed the identity of S22 (**Figure 4A**, lane 4).

The gene for the DNA vaccine, supplied by GenScript, was subcloned into the pVAX expression vector, generating the pVS22 DNA vaccine construct (4500 bp) (**Figure 4B**, lane 2). The correct cloning of pVS22 was verified via agarose gel electrophoresis, which revealed two fragments: one approximately 1500 bp, corresponding to the gene of the S22 protein, and the other around 3000 bp, corresponding to the pVAX vector (**Figure 4B**, lane 3).

### 3.5 Evaluation of the humoral immune response

The humoral response was assessed by measuring anti-S22 IgG1 and IgG2a antibody levels in the different experimental and control groups. At 15 and 30 days post-immunization (pi), significant production of IgG1 was observed in mice immunized with the recombinant S22 protein. In contrast, mice immunized with the pVS22 or the DNA prime-protein boost strategy exhibited antibody levels comparable to pre-immune animals. Notably, at 45 days pi, mice immunized with S22 protein, and the DNA prime-protein boost strategy showed significantly higher IgG1 levels (*P* < 0.0001) than the negative control groups (PBS or pVAX). The pVS22 DNA construct did not elicit IgG1 production throughout the experimental period. Both the S22 protein and pVS22 vaccinations elicited high levels of IgG2a at 15 days pi. However, the DNA prime-protein boost technique did not elicit high antibody levels by 30 days pi. Mice immunized with the S22 protein, and the DNA prime-protein boost strategy showed significant IgG2a levels. At 45 days pi, all the immunizations (S22 protein, pVS22, and DNA prime-protein boost) induced significantly higher IgG2a levels than the control groups (**Figure 4C**).

### 3.6 Evaluation of the cellular immune response

Cell-mediated immunity was evaluated by stimulating lymphocytes with the multivalent recombinant protein S22 and *B. abortus* total proteins. Splenocytes from mice immunized with the S22 protein showed significant proliferation compared to the controls (PBS and pVAX) when stimulated with 1 μg/ml and 5 μg/ml of S22 (P < 0.05) and with 4 μg/ml and 20 μg/ml of *B. abortus* total proteins (P < 0.001 and P < 0.0001, respectively). Similarly, splenocytes from mice immunized with the pVS22 DNA construct exhibited significant proliferation compared to the negative controls when stimulated with 5 μg/mL of recombinant S22 protein (P < 0.01), as well as with 4 μg/ml and 20 μg/ml of crude *B. abortus* proteins (P < 0.0001). In contrast, in mice immunized using the DNA prime-protein boost strategy, significant proliferation was observed only when splenocytes were stimulated with 20 μg/mL of crude *B. abortus* protein (P < 0.001) (**Figure 4D**).

### 3.7 Cytokine secretion

Regarding cytokine production, a significant increase in IFN-γ was noted in splenocytes from animals immunized with the S22 protein and the DNA prime-protein boost strategy when stimulated with 5 μg/ml of recombinant S22 protein (P < 0.0001). However, no significant differences in IL-4 and TNF-α production were observed under any concentration of the S22 protein (**Figure 4E**).

### 3.8 Protection assay

The results of the protection assay conducted post-challenge with 1x10^4 CFU of *B. abortus* 2308 indicated that the S22 protein, the pVS22 DNA construct, and the DNA prime-protein boost strategy did not confer significant protection compared to the negative control mice. Conversely, the *B. abortus* RB51 vaccine strain, which is a positive control for protective efficacy, induces significant protection levels of 1.18 protection units (**Table 2**).

**Table 2:** Protection conferred by S22, pVS22 and prime boost strategy vaccination BALB/c mice challenged with *B. abortus* 2308 strain.

Experimental groups	Log10 *B. abortus* CFU per spleen (mean +/-SD)	Protection Units
PBS	3.37 +/- 0.15	0
pVAX	3.07 +/- 0.20	0.298
pVS22	3.22 +/- 0.31	0.149
S22	2.87 +/- 0.42	0.499
Prime-Boost	3.16 +/- 0.05	0.213
*B. abortus* RB51	2.19 +/- 0.51	1.18*

(*) shows significant differences (P < 0.05).

## 4 Discussion

In this study, we designed a multivalent vaccine candidate against *B. abortus*, taking advantage of the immunogenic potential of three selected ORFs from *Brucella* (16). Our engineered construct, depicted in **Figure 2A** and called S22, underwent extensive *in silico* analyses to evaluate its physicochemical properties, antigenicity, allergenicity, and toxicity. Using bioinformatic tools (SOLpro), we ascertained that S22 exhibits favorable solubility and hydrophobicity characteristics, essential for efficient expression and purification in *E. coli*. Furthermore, evaluations using the VaxiJen v2.0 server and ANTIGENpro suggest that S22 is highly immunogenic. ToxinPred2 classified S22 as non-toxic, and AllerTOP v.2.0 server data suggests it is non-allergenic (**Table 1**). These comprehensive analyses underscore that our designed S22 protein is not only immunogenic but also viable for production and safe for host administration. The efficacy of a vaccine in eliciting a stable immune response depends on its interaction with receptors of immune cells (36). TLR4, a pathogen recognition receptor expressed in several types of immune cells, including monocytes, macrophages, and dendritic cells, plays a pivotal role in this process (37). Unlike other TLRs that assemble on the cell surface, a subset of TLR4- lymphocyte antigen 96 (MD2)-LPS complexes can be recruited to an endosomal compartment to activate an alternative signal transduction pathway for the induction of proinflammatory cytokines and type I interferons (IFNs) (38). The main agonist of TLR4 is LPS; however, other ligands, such as proteins or synthetic agonists different from LPS, can interact with this receptor (39, 40). Recognizing this, we explored the binding affinity of S22 to TLR4. Our approach involved constructing a 3D model of the tertiary structure of S22 using the Intfold7 server, recently enhanced with cutting-edge deep learning methods for accurate protein structure prediction (24). This model, subsequently refined with GalaxyRefine, showed energetically favorable and high-quality characteristics similar to native proteins clarified through X-ray crystallography. Validation by the ERRAT server confirmed the superior quality of the model, with a quality factor higher than 97.5, indicating a highly reliable structure (41) (**Figure 2**).

The refined S22 model and the crystallized TLR4/MD2 complex from the PDB database were subjected to a molecular docking analysis. This revealed that S22 engages favorably with the TLR4/MD2 complex at three distinct sites. A subsequent molecular dynamic simulation (MDS), a critical step for assessing vaccine stability under *in vivo* conditions (36), predicted the stabilization of the S22 protein, both in its free form and in conjunction with the TLR4/MD2 complex, at 15 ns. A lower RMSF value indicates less flexibility and higher stability (42). High RMSF values associated with regions of the linker and SH3 domain-like protein region of the S22 vaccine were reduced by forming the S22-TLR4/MD2 complex, suggesting a stable interaction. This interaction is crucial for activating the innate immune response and, in turn, potentiating the adaptive immune response.

In this work, we also evaluated the immune response triggered by vaccinating mice with the S22 protein, the pVS22 DNA construct, and the DNA prime-protein boost strategy. The use of different immunization routes is mainly based on the fact that subunit-based vaccines, such as the S22 protein, are administered with some type of adjuvant and the route of administration depends on the adjuvant. In the case of CFA, this is normally administered i.d. and IFA by s.c. or i.p. On the other hand, DNA vaccines have always been administered i.m (16). That is why in the primary immunization group with DNA/protein booster, the DNA doses were administered i.m. and the protein dose i.d. The immunization with S22 protein induced robust levels of both IgG1 and IgG2a, suggesting the activation of a comprehensive and balanced Th1 and Th2 immune response. The administration of the pVS22 DNA construct elicited a marked increase in IgG2a antibodies, a marker typically indicative of Th1 responses in mice (43). Notably, a balanced immune response was observed in the DNA prime-protein boost group. In this case, the initial responses were similar to those observed in the pVS22 group; however, with the third immunization with S22, the immune response evolved towards a mixed response characterized by higher production of both IgG1 and IgG2a. We also observed that antigen-specific splenocytes from mice immunized with either the S22 protein or pVS22 DNA construct displayed significant proliferation upon stimulation with the S22 protein, surpassing the response of the control groups. Intriguingly, the pVS22 group required higher protein concentrations for optimal lymphoproliferation than the S22 group. This finding suggests differential sensitivities in immune cell activation between the two vaccine candidate formulations. Additionally, splenocytes from all immunized mice exhibited significant proliferation when stimulated with *B. abortus* total proteins. Under the evaluated conditions, splenocytes from the S22 protein and DNA prime-protein boost groups secreted elevated levels of IFN-γ. The importance of IFN-γ, especially in orchestrating macrophage bactericidal activity and favoring the production of protective IgG2a antibodies, cannot be overstated in the context of immunity against *B. abortus* (44).

The potential of a heterologous DNA prime-protein boost strategy has been primarily explored in the context of viral pathogens, including COVID-19, Tembusu virus, HIV, and hepatitis C virus (45–48), with emerging applications against bacterial pathogens like *Leptospira* spp, *Mycobacterium tuberculosis*, and *Mycobacterium kansassi* (49, 50). In our study, while the DNA prime-protein boost did not significantly enhance overall protective levels, it effectively modulated antibody production from a predominantly IgG2a response to a balanced IgG1 and IgG2a profile. Moreover, this strategy successfully stimulated lymphoproliferation against *B. abortus* total proteins and promoted IFN-γ production, an outcome not achieved solely with the DNA vaccine. This underscores the nuanced yet critical role of vaccine strategies in shaping immune responses, a factor that could be pivotal in developing more effective interventions against *B. abortus*.

Immunoinformatic tools made it possible to generate a new multivalent vaccine candidate against *B. abortus.* Administered intramuscularly and/or intradermally, this vaccine candidate demonstrated significant immunogenicity. Although it failed to elicit a substantial protective response against the pathogen, our results underscore the utility of the *in silico* design. This approach effectively predicted and optimized expression, purification, and immune response induction against customized antigens, significantly reducing the development time and costs. Such advantages align with those reported in the design of vaccines for other pathogens, including SARS-CoV-2 (51).

The licensed RB51 vaccine, used as a positive control in our study and known for inducing strong cellular immunity, which predominantly stimulates cell-mediated immunity (Th1 type responses), conferred a high level of protection against *B. abortus* (**Table 2**). Interestingly, our findings using specifically S22, induced high levels of immunogenicity but low levels of protection, demonstrating a discrepancy between our experimental vaccines and *B. abortus* RB51, which suggests that the protection against *Brucella* involves a complicated balance between Th1/Th2- immune response (52, 53). Moreover, these experimental vaccines (S22 and pVS22) induced lower immunogenicity and protective response compared to the SOD-only vaccine (15). Thus, we hypothesize that this effect is due to competition among epitopes from SOD, ZnMP and SH3 proteins, which could specifically reduce (ZnMP and SH3 protein epitopes) the immune response conferred by SOD, recognized as the most immunogenic protein used in the construction of our experimental vaccines against *B. abortus*. Our results demonstrate that the same antigenic construct can elicit different immune responses, depending on its formulation, either as a purified protein (S22) or as a gene in a DNA vector (pVS22). In addition, our study illustrates the potential of the DNA prime-protein boost strategy in modulating immune responses. This immunization scheme shifted the pVS22-induced response, characterized by IgG2a production without IFN-γ, towards a mixed Th1/Th2 response with IFN-γ production, similar to the response induced by the S22 protein alone. Therefore, these findings open up new avenues for vaccine development and underscore the importance of careful antigen selection in the design of multivalent vaccines. The combination of antigens can lead to suboptimal immune responses due to potential antagonism or competition between epitopes, which may reduce the vaccine efficacy, an important aspect that must be considered in future studies.

## Data Availability

The original contributions presented in the study are included in the article/supplementary materials, further inquiries can be directed to the corresponding author/s.
